# RETRACTED: Frolli et al. The Impact of COVID-19 on Cognitive Development and Executive Functioning in Adolescents: A First Exploratory Investigation. *Brain Sci.* 2021, *11*, 1222

**DOI:** 10.3390/brainsci16080826

**Published:** 2026-08-04

**Authors:** Alessandro Frolli, Maria Carla Ricci, Francesca Di Carmine, Agnese Lombardi, Antonia Bosco, Emilio Saviano, Luisa Franzese

**Affiliations:** 1 Disability Research Centre, University of International Studies in Rome, 00147 Rome, Italy; m.ricci@unint.eu (M.C.R.); f.dicarmine@unint.eu (F.D.C.); lombardiagnese@gmail.com (A.L.); ant.bosco@hotmail.it (A.B.); 2 FINDS-Italian Neuroscience and Developmental Disorders Foundation, 81040 Caserta, Italy; 3 Regional School Office, 80133 Napoli, Italy; luisa.franzese@istruzione.it

The journal retracts the article titled “The Impact of COVID-19 on Cognitive Development and Executive Functioning in Adolescents: A First Exploratory Investigation” [1], cited above.

Following publication, concerns were raised with the Editorial Office regarding the statistical analysis and ethical approval information reported in this publication [1]. In accordance with the journal’s standard procedures, the Editorial Office and Editorial Board conducted an investigation during which questions were raised regarding certain parts of the statistical analysis and the lack of appropriate Institutional Review Board approval, prior to the participant enrolment.

The authors cooperated with the investigation and proposed a Correction, providing corrected statistical results and additional information relating to the ethical approval. However, after careful consideration, the Editorial Board determined that the explanations and supporting documentation did not sufficiently address the remaining concerns about the ethical approval information and that the study is non-compliant with MDPI’s policies for Research Involving Human Subjects. 

As a result, the Editorial Board has decided to retract this paper as per MDPI’s retraction policy. 

This retraction was approved by the Editor-in-Chief of the *Brain Sciences* journal. 

The authors have been informed of this retraction and have indicated their disagreement with this decision. 
